# Significant artefactual noise in ^90^Y TOF-PET imaging of low specific activity phantoms arises despite increased acquisition time

**DOI:** 10.1186/s40658-019-0259-6

**Published:** 2019-11-28

**Authors:** Michel Hesse, Stephan Walrand

**Affiliations:** Universite Catholique de Louvain, Brussels, Brabant Belgium

## Abstract

Volumes of usual PET phantoms are about four to sixfold that of a human liver. In order to avoid count rate saturation and handling of very high ^90^Y activity, reported TOF-PET phantom studies are performed using specific activities lower than those observed in liver radioembolization.

However, due to the constant random coincidence rate induced by the natural crystal radioactivity, reduction of ^90^Y specific activity in TOF-PET imaging cannot be counterbalanced by increasing the acquisition time. As a result, most ^90^Y phantom studies reported images noisier than those obtained in whole-body ^18^F-FDG, and thus advised to use dedicated noise control in TOF-PET imaging post ^90^Y liver radioembolization.

We performed acquisitions of the Jaszczak Deluxe phantom in which the hot rod insert was only partially filled with 2.6 GBq of ^90^Y. Standard reconstruction parameters recommended by the manufacturer for whole-body ^18^F-FDG PET were used.

Low specific activity setups, although exactly compensated by increasing the acquisition time in order to get the same number of detected true coincidences per millilitre, were impacted by significant noise. On the other hand, specific activity and acquisition time setup similar to that used in post ^90^Y liver radioembolization provided image quality very close to that of whole-body ^18^F-FDG.

This result clearly discards the use of low specific activity phantoms intended to TOF-PET reconstruction parameter optimization. Volume reduction of large phantoms can be achieved by vertically setting the phantoms or by adding Styrofoam inserts.

## Background

The unusual 32 positron emissions per million decays and the speckled pattern observed in ^90^Y TOF-PET liver images initiated the widespread paradigm that this modality requires dedicated noise control. This belief was consolidated by many studies using large volume phantoms involving low specific activities to avoid PET saturation resulting in doses as low as 5 Gy for some of them. In addition, the acquisition time increase was often too limited to get the same number of detected true coincidences per milliliter as in post ^90^Y liver radioembolization.

However, due to the constant random rate originating from the L[Y]SO crystal radioactivity jointly with the low ^90^Y positron yield, lower specific ^90^Y activities cannot be counterbalanced by increased acquisition times [1]. Moreover, Monte Carlo simulations [2], ex vivo autoradiography [3] and ^166^Ho MRI [4] proved the speckled nature of the sphere distribution in the liver.

## Phantom presentation

**Fig. 1:**
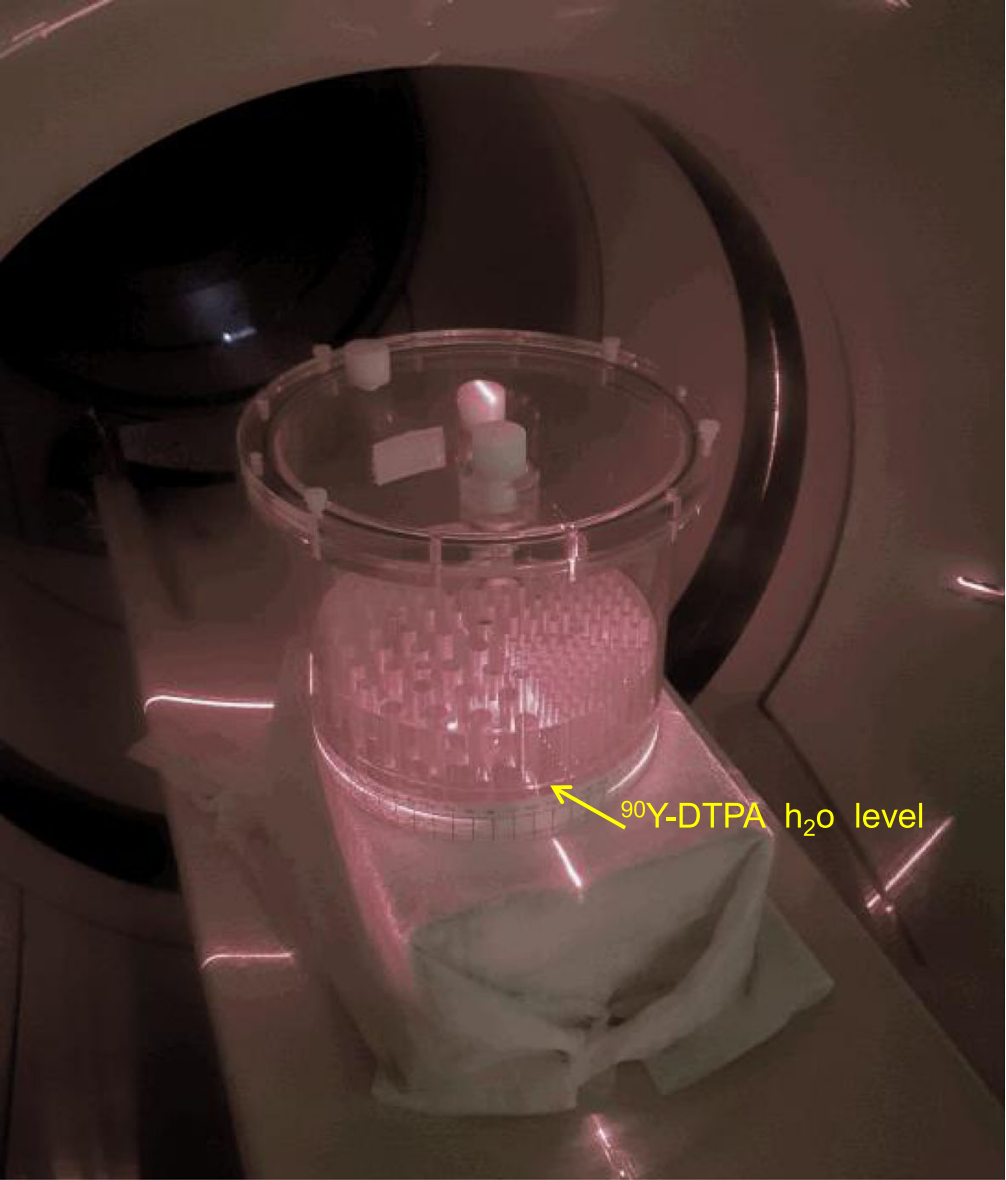
Jaszczak deluxe phantom set in vertical position on a 20-cm-thick paper bloc modelling the conventional attenuation as only a part of the hot rod insert was filled with active solution in order to reach a typical clinical liver absorbed dose in selective liver ^90^Y radioembolization

A Jaszczak Deluxe phantom was set vertically (Fig. 1). The hot rod insert (inner rods diameters 4.8, 6.4, 7.9, 9.5, 11.1, 12.7 mm) was partially filled with different solutions: first, with a ^18^F-FDG solution corresponding to a mean liver SUV = 2 in a 300-MBq 75-kg patient study; secondly, with a 2.65-GBq ^90^Y—300 ml DTPA water solution, giving a 18-mm height filling of the rods, which resulted in *a* ≈ 100 Gy mean absorbed dose in each filled sector slices. A 20-cm-thick attenuating medium, set under the phantom, modelled the transverse attenuation observed for the fully filled phantom set in conventional horizontal position.

Two bed positions were acquired, and the times per bed position were 1.5 min for ^18^F and 20 min for ^90^Y, respectively. The ^90^Y phantom was further imaged for 200 min per bed position after a 10- and 20-fold factor decaying; for this last setup, two slices were summed together to exactly compensate the activity reduction. The Philips Gemini TF64 system had a TOF-fwhm of 550 ps. Standard FDG reconstruction parameters were used for all the acquisitions, i.e. 3 iterations × 33 subsets.

**Fig. 2:**
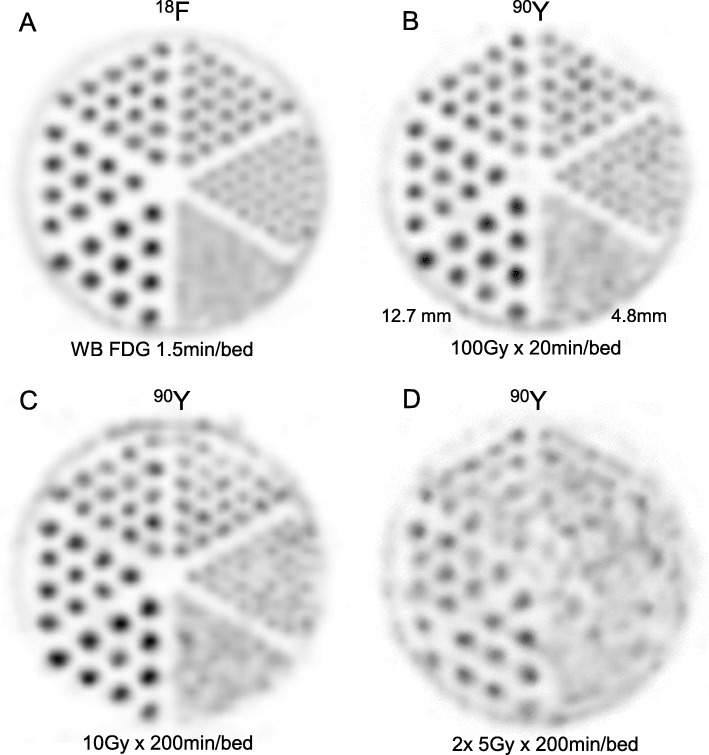
**a**, **b** ^18^F and ^90^Y hot rod sector slices obtained using specific activities and acquisition times similar to that of clinical whole-body ^18^F-FDG and of post ^90^Y liver radioembolization, respectively. **c**, **d** ^90^Y hot rod sector slices obtained using lower specific activities compensated by longer acquisition times, and additionally in **d** by slice summation, in order to get the same number of detected coincidences per millilitre

Hot rods were individually visualized in five sectors for the ^18^F setup and for the 100 Gy × 20 min/bed-^90^Y setup as well; this later setup was affected only by a little higher noise level (Fig. 2). A clear degradation of the hot rods intensities was observed for the 6.4, 7.9, and 9.5 mm rods in the 10 Gy × 200 min/bed setup while all sectors were dramatically impacted in the 2 × 5 Gy × 200 min/bed despite the exact acquisition time compensation in both setups.

## Conclusion

Despite a slightly higher noise level, the spatial resolution of the ^90^Y 100 Gy setup image using standard reconstruction parameters was very close to that of the WB ^18^F-FDG setup, explaining the recent success of TOF-PET-based EUD in dose-response prediction [5].

Although time acquisition compensated, the 10 Gy and 5 Gy setup images were impacted by significant artefactual noise which clearly rules out the use of low specific activity phantom setups intended to reconstruction parameter optimization.

Volume reduction of large phantoms can also be achieved by vertically setting the phantoms or by adding Styrofoam inserts [6]. The phantom mean absorbed dose should always be reported.

## Data Availability

There are available.
